# Gaps in the type 2 diabetes care cascade: a national perspective using South Africa’s National Health Laboratory Service (NHLS) database

**DOI:** 10.1186/s12913-023-10318-9

**Published:** 2023-12-21

**Authors:** Alana T. Brennan, Evelyn Lauren, Jacob Bor, Jaya A. George, Kamy Chetty, Koleka Mlisana, Andrew Dai, Siyabonga Khoza, Sydney Rosen, Andrew C. Stokes, Frederick Raal, Patricia Hibberd, Sara M. Alexanian, Matthew P. Fox, Nigel J. Crowther

**Affiliations:** 1 Department of Global Health, Boston University School of Public Health, Boston, MA USA; 2 Health Economics and Epidemiology Research Office, Faculty of Health Sciences, University of the Witwatersrand, Johannesburg, South Africa; 3 Department of Epidemiology, Boston University School of Public Health, Boston, MA USA; 4 Department of Biostatistics, Boston University School of Public Health, Boston, MA USA; 5 Wits Diagnostic Innovation Hub, University of the Witwatersrand, Johannesburg, South Africa; 6 National Health Laboratory Service, Johannesburg, South Africa; 7 Academic Affairs, Research & Quality Assurance, National Health Laboratory Service, Johannesburg, South Africa; 8 Department of Mathematics and Statistics, Boston University, Boston, USA; 9 Department of Chemical Pathology, University of the Witwatersrand, Faculty of Health Sciences, Johannesburg, South Africa; 10 Division of Endocrinology and Metabolism, Department of Internal Medicine, Faculty of Health Sciences, University of the Witwatersrand, Johannesburg, South Africa; 11 Boston University School of Medicine, Boston, MA USA

**Keywords:** Type 2 diabetes mellitus, HIV/AIDS, Tuberculosis, South Africa, Care cascade

## Abstract

**Background:**

Research out of South Africa estimates the total unmet need for care for those with type 2 diabetes mellitus (diabetes) at 80%. We evaluated the care cascade using South Africa’s National Health Laboratory Service (NHLS) database and assessed if HIV infection impacts progression through its stages.

**Methods:**

The cohort includes patients from government facilities with their first glycated hemoglobin A1c (HbA1c) or plasma glucose (fasting (FPG); random (RPG)) measured between January 2012 to March 2015 in the NHLS. Lab-diagnosed diabetes was defined as HbA1c ≥ 6.5%, FPG ≥ 7.0mmol/l, or RPG ≥ 11.1mmol/l. Cascade stages post diagnosis were retention-in-care and glycaemic control (defined as an HbA1c < 7.0% or FPG < 8.0mmol/l or RPG < 10.0mmol/l) over 24-months. We estimated gaps at each stage nationally and by people living with HIV (PLWH) and without (PLWOH).

**Results:**

Of the 373,889 patients tested for diabetes, 43.2% had an HbA1c or blood glucose measure indicating a diabetes diagnosis. Amongst those with lab-diagnosed diabetes, 30.9% were retained-in-care (based on diabetes labs) and 8.7% reached glycaemic control by 24-months. Prevalence of lab-diagnosed diabetes in PLWH was 28.6% versus 47.3% in PLWOH. Among those with lab-diagnosed diabetes, 34.3% of PLWH were retained-in-care versus 30.3% PLWOH. Among people retained-in-care, 33.8% of PLWH reached glycaemic control over 24-months versus 28.6% of PLWOH.

**Conclusions:**

In our analysis of South Africa's NHLS database, we observed that 70% of patients diagnosed with diabetes did not maintain in consistent diabetes care, with fewer than 10% reaching glycemic control within 24 months. We noted a disparity in diabetes prevalence between PLWH and PLWOH, potentially linked to different screening methods. These differences underscore the intricacies in care but also emphasize how HIV care practices could guide better management of chronic diseases like diabetes. Our results underscore the imperative for specialized strategies to bolster diabetes care in South Africa.

**Supplementary Information:**

The online version contains supplementary material available at 10.1186/s12913-023-10318-9.

## Background

Type 2 diabetes mellitus (diabetes) is an escalating public health crisis in South Africa. From 2016 to 2018, diabetes was the 2^nd^ leading cause of death in the country, responsible for 6% of overall deaths [1]. When stratified by sex, diabetes was the leading underlying cause of death for females (7.7% of overall deaths) and 4^th^ leading cause for males (4.4% of overall deaths) [1]. In March 2022, the International Diabetes Federation estimated South Africa’s adult diabetes prevalence at 11.3%, up from 9.0% in 2015 [2]. In Sub-Saharan Africa, the current control of diabetes and cardiovascular risks is insufficient, leading to complications that strain healthcare systems. Efforts to improve care are hindered by a lack of understanding of the deficiencies in the existing care continuum.

Adherence to the recommended guidelines for treating individuals with diabetes in low- and middle-income countries (LMICs), South Africa included, is estimated to be poor [3, 4]. LMICs continue to face hurdles in identifying and applying the techniques needed to effectively carry out what is known as the diabetes care cascade. The diabetes care cascade is a multifaceted approach designed to provide continuous and effective management of diabetes. It consists of several interconnected stages, beginning with the early detection and diagnosis of the disease, followed by the initiation of appropriate treatment, and finally, ongoing management and control to maintain optimal health [5]. This continuum requires seamless integration and collaboration among healthcare providers, educators, and patients, and any disruption or failure in one stage can lead to suboptimal outcomes. In LMICs, achieving this integrated approach presents significant challenges, often resulting in gaps in care and unmet needs for patients [3, 4]. A recent cross-sectional analysis involving data from 28 LMICs, inclusive of 10 African nations and specific data from South Africa, revealed significant shortcomings in the health system's handling of diabetes care [3]. The study highlighted substantial gaps at the diabetes testing phase and low success rates in controlling the disease, culminating in an estimated 77% total unmet need for comprehensive diabetes care [3].

South Africa continues to grapple with ongoing epidemics of HIV and tuberculosis. In the past decade, a concerted effort has been made to utilize the existing clinical tools, strategies, and systems developed for HIV in the fight against non-communicable diseases like diabetes in LMICs [6, 7]. This integration into the healthcare system might lead to those being treated for HIV having a higher chance of being screened, diagnosed, and treated for diabetes when compared to the general population. While more research is required to substantiate this hypothesis, findings from prior studies hint at the potential for HIV care and treatment programs to provide a foundational framework for broader population health improvements through enhanced programmatic integration. The relatively robust care for HIV and tuberculosis in South Africa, compared to non-communicable disease care, might present valuable opportunities for improved non-communicable disease monitoring and management [6, 7].

South Africa’s overlapping epidemics of diabetes, HIV, and tuberculosis pose a serious health and economic burden to the country’s already overburdened and under-resourced health system. Understanding the relationships among these diseases in individuals with multiple morbidities in an under-resourced environment is critical. We therefore used data from South Africa’s National Health Laboratory Service (NHLS) database from 2012-2017 to assess the prevalence of laboratory-diagnosed diabetes and unmet need for care in public-sector health facilities. We estimated the share of people diagnosed with diabetes who were retained-in-care (based on diabetes testing) and achieved glycaemic control by 24-months post diagnosis. These estimates were conducted nationally and categorized by HIV and tuberculosis status., shedding light on the complex challenges and opportunities for target care within these overlapping health crises.

## Methods

### Study population

Patients eligible for our study were those who had their first reported blood glucose (random/fasting) or HbA1c between January 1, 2012-March 31, 2015. We chose this interval because, prior to 2012, South Africa's primary health focus was on addressing the HIV and tuberculosis epidemics, with less emphasis on non-communicable disease screening and because the dataset was last updated for non-communicable diseases laboratory tests on April 1, 2017. All patients had the potential for 24 months of follow-up to assess care cascade outcomes. We further restricted the analysis to patients ≥ 30 years old at first blood glucose or HbA1c to best exclude those with type 1 diabetes mellitus. We were unable to rule out late-onset autoimmune diabetes, a subtype of type 1 diabetes diagnosed after age 30. As we did not have pregnancy status for females, we cannot rule out the presence of subjects with gestational diabetes in our cohort.

### NHLS cohort creation and description

South Africa’s NHLS is the sole provider of laboratory services for the public sector health system, which serves 80% of the population across all provinces [8]. The study cohort was assembled using an innovative data linkage method as detailed in previous literature [9]. Briefly, while data from HIV-related laboratory tests were gathered over the years, various inaccuracies emerged due to administrative errors and patient relocations, leading to inconsistencies in patient records. To rectify this, we developed a record linkage algorithm to identify unique patients in the NHLS database, combining elements of probabilistic linkage approaches with concepts from network analysis. The matching process was informed by available demographic data for each specimen, including first name, last name, date of birth, gender, province, and medical facility. The algorithm was developed and validated for application to 35 million CD4 counts and viral loads. To validate the algorithm, 59,000 potential matches from a sample of 1,000 specimens underwent manual review. The algorithm's efficacy, gauged through sensitivity and other evaluation metrics, was compared with techniques such as exact matching and a pre-existing identifier from the NHLS Corporate Data Warehouse. The algorithm attained a sensitivity of 93.7% and positive predictive values (PPV) of 98.6% [9]. In 2019, the record-linking methods were extended to all HIV, tuberculosis, and non-communicable disease laboratory tests to create the NHLS Multi-morbidity Cohort for this analysis. We conducted a manual assessment on around 7,000 non-communicable disease lab results related to 250 patients. The evaluation showed a PPV of 95.1%, comparable to what was observed solely in the HIV cohort, although there was a reduced sensitivity of 78.3%. While we expected a minor decline in performance when broadening the algorithm to include a greater number of labs, we were content with the elevated PPV, as it is the most vital measure. This high PPV gives us confidence that the algorithm is 95% accurate in identifying a match when it does so.

Currently, the NHLS Multi-morbidity Cohort has more than 68 million laboratory measurements corresponding to 30 million unique patients (≥ 16 years old) with at least one laboratory measurement between April 1, 2004-March 31, 2017. It contains a unique anonymized patient identifier, biological sex, age, laboratory test date, test type, test result, health facility, district, and province.

### Diabetes guidelines

During the study period, the Society for Endocrinology, Metabolism and Diabetes of South Africa (SEMDSA) 2012 guidelines were adopted in public-sector health facilities across South Africa [10]. These guidelines not only endorsed the use of HbA1c for screening and diagnosing diabetes but also highlighted its significance in assessing glycemic control. Furthermore, the guidelines also recommend random and fasting blood glucose tests as alternative diagnostic methods. We recognize that, in practical scenarios, clinicians may have favored point-of-care testing as an alternative to formal laboratory examinations when evaluating glycemic control, often influenced by factors such as immediate accessibility and faster results. However, in contrast to this inclination, prior research indicates that point-of-care testing was infrequently employed (4%) in comparison to formal laboratory tests (96%) [11]. This observation was made in a substantial sample of over 500,000 individuals attending government sector clinics in the Gauteng province during a comparable timeframe to our study [11].

Diabetes screening methods can be categorized into three distinct approaches: i) random screening, applies to low-risk individuals who undergo glucose testing incidentally on individuals without traditional risk factors or symptoms of diabetes; ii) opportunistic screening, used to identify as high-risk during consultations for unrelated health matters, particularly for individuals with known risk factors (i.e., obesity or a family history of diabetes); and iii) targeted screening of individuals due to their high-risk factors and specific symptoms, such as excessive thirst or frequent urination [10]. SEMDSA guidelines require diagnosis of diabetes be based on formal laboratory testing and not point-of-care instruments [10]. It is important to note that we were unable to identify the reason for testing in our cohort.

### Outcomes

**Fig. 1:**
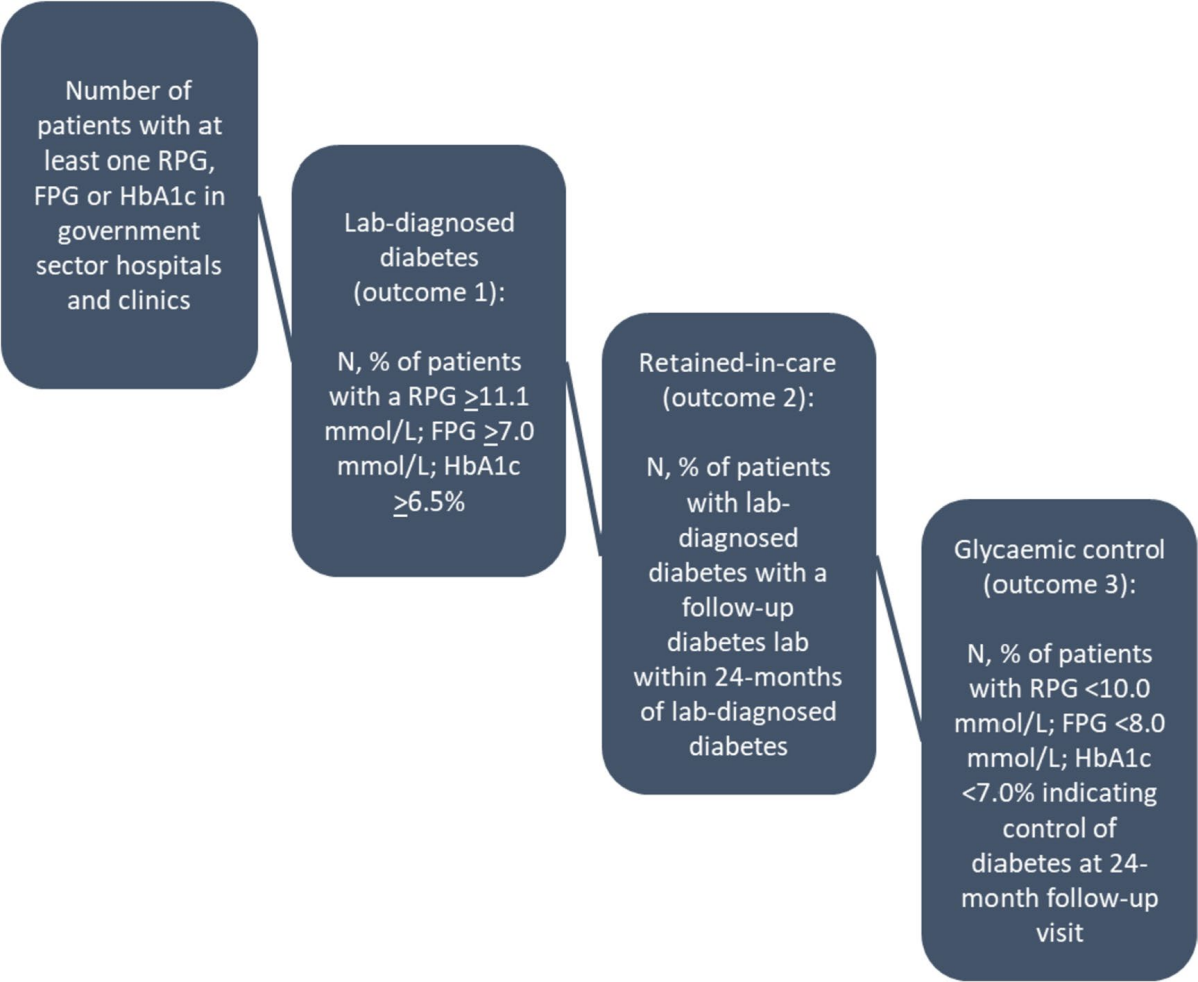
The diabetes care cascade based on laboratory data [10]

Study outcomes are drawn from the diabetes cascade of care, illustrated in Fig. 1. We defined three sequential, primary outcomes for the study, as shown below:

1. *Lab-diagnosed diabetes:* one elevated HbA1c ≥ 6.5%; or fasting glucose ≥ 7.0mmol/l; or random glucose ≥ 11.1mmol/l [10].
2. *Retained-in-care based on diabetes lab tests:* an HbA1c, fasting glucose, or random glucose at least one-month post lab-diagnosis up to 24 months of the first diabetes lab. (*note*: we also broaden the definition of retained-in-care to encompass all laboratory tests conducted within 24 months following a diabetes diagnosis—not just those specific to diabetes—to assess if patients were accessing care for other reasons.)
3. *Glycaemic control:* an HbA1c < 7.0%; or fasting glucose < 8.0mmol/l; or random glucose < 10.0mmol/l within 24-months of elevated glucose or HbA1c [10].

Guidelines recommend that HbA1c be used to assess glycaemic control when possible but allow the choice between HbA1c and glucose to be based on local considerations [10]. As such, we allowed use of plasma fasting or random blood glucose to indicate glycaemic control.

Our three outcomes are aligned with discrete steps along the diabetes cascade of care*.* To quantify the cascade, we identified the number of unique patients tested for diabetes and then calculated the proportion of patients: 1) who met lab-diagnostic criteria for diabetes (outcome 1); 2) retained-in-care based on diabetes lab tests (outcome 2); and 3) demonstrated glycaemic control at the outcome 2 follow-up lab (outcome 3). Results were stratified by facility type, HIV status (defined as patients with an HIV-associated test – CD4 count, HIV viral load or positive HIV test – any time prior to first blood glucose or HbA1c up to 24 months after, and acute tuberculosis infection (defined as patients with a tuberculosis-associated test (i.e. culture, smear, first-line probe assay, drug susceptibility tests, GeneXpert, polymerase chain reaction) 6 months prior to blood glucose or HbA1c up to 6 months after). As results did not differ by province, we display results at the national level.

Estimates of all outcomes were age-standardized to the age distribution of the South African adult population using mid-year population estimates for 2021 [12]. Age-standardization was carried out using five-year age-categories between 30–74 and an open-ended category of ≥ 75.

### Data analysis

We fit three mixed effects modified Poisson regression models [13] to determine predictors of all three outcomes listed above. Facilities nested within districts were included as random effects to control for within-group homogeneity for districts and individual health facilities. In the models we controlled for HIV status, acute tuberculosis infection, province, facility type (hospital or clinic based on location of first blood glucose/HbA1c performed), year of first diabetes lab (2012–2015), sex, and age (30–39.9, 40–49.9, 50–59.9 and ≥ 60 years). For the model assessing predictors of lab-diagnosed diabetes and glycemic control we also included the type of diagnostic used (e.g., HbA1c, fasting or plasma glucose).

## Results

### Cohort description and testing trends

A total of 373,889 patients were tested for diabetes in a hospital or clinic (Table 1) from January 1, 2012 to March 31, 2015. Median age at first test was 54 years (interquartile range (IQR):44–64 years). Patients were predominately female (63.5%) and the majority (62.0%) had their first blood glucose or HbA1c conducted in a hospital. Most were living without HIV (84.8%) or acute tuberculosis infection (98.5%). Sex, facility type, HIV and tuberculosis distributions were similar across all nine provinces. Two exceptions were the Eastern Cape and North West Province, where there were more equal proportions of patients tested in hospitals and clinics.

**Table 1:** Demographic and clinical characteristics of the cohort at first blood glucose or HbA_1c_ stratified by province (*N* = 373,889)

Characteristic	Measure	GP (*n* = 118,194, 31.6%)	KZN (*n* = 73,617, 19.7%)	WC (*n* = 28,742, 7.7%)	EC (*n* = 54,931, 14.7%	FS (*n* = 24,258, 6.5%)	LP (*n* = 17,464, 4.7%)	MP (*n* = 25,438, 6.8%)	NC (*n* = 7,775, 2.1%)	NW (*n* = 23,470, 6.3%)	Total (*N* = 373,889)
**Female**	**n (%)**	73,174 (61.9%)	48,514 (65.9%)	17,134 (59.6%)	36,888 (67.2%)	14,517 (59.8%)	10,794 (61.8%)	16,365 (64.3%)	5,178 (66.6%)	14,862 (63.3%)	237,426 (63.5%)
**Age (years)**	**median (IQR)**	54 (43, 64)	55 (45, 64)	51 (41, 61)	56 (46, 65)	53 (41, 63)	55 (45, 66)	55 (46, 65)	51 (40, 62)	55 (46, 65)	54 (44, 64)
**FBG (mmol/l)**	**median (IQR)**	5.5 (4.6, 8.7)	5.8 (4.9, 8.3)	5.6 (4.9, 7.5)	6.8 (5.1, 11.3)	5.8 (4.7, 9.0)	7.5 (5.1, 12.7)	7.5 (5.3, 13.2)	5.5 (4.7, 8.3)	6.8 (5.1, 12.6)	5.84 (4.8, 9.1)
**RBG (mmol/l)**	**median (IQR)**	5.6 (4.7, 8.6)	5.8 (4.9, 7.9)	5.5 (4.8, 7.4)	6.3 (4.9, 11.8)	5.6 (4.6, 7.4)	7.6 (5.2, 18.0)	6.9 (5.0, 14.3)	5.5 (4.6, 8.2)	6.5 (4.9, 13.0)	5.8 (4.8, 8.9)
**HbA**_**1c**_** (%)**	**median (IQR)**	7.1 (6.0, 9.9)	7.8 (6.2, 10.4)	7.0 (5.9, 9.2)	8.5 (6.4, 11.2)	7.1 (5.8, 9.9)	9.6 (6.9, 13.0)	7.9 (6.0, 11.0)	6.7 (5.8, 9.2)	7.4 (6.0, 10.8)	7.6 (6.1, 10.6)
**Lab-diagnosed diabetes**	**n (%)**	55,086 (46.6%)	42,821 (58.2%)	6,051 (21.1%)	33,573 (61.1%)	6,967 (28.7%)	10,748 (61.5%)	15,163 (59.6%)	3,112 (40.0%)	13,143 (56.0%)	186,664 (49.9%)
**Lab-diagnosed pre-diabetes**	**n (%)**	958 (0.8%)	560 (0.8%)	64 (0.2%)	459 (0.8%)	183 (0.8%)	100 (0.6%)	367 (1.4%)	64 (0.8%)	152 (0.6%)	2,907 (0.8%)
**HIV status**	**HIV + **	18,488 (15.6%)	9,995 (13.6%)	4,254 (14.8%)	5,978 (10.9%)	5,605 (23.1%)	2,502 (14.3%)	4,864 (19.1%)	1,238 (15.9%)	3,805 (16.2%)	56,729 (15.2%)
	**HIV-**	99,706 (84.4%)	63,622 (86.4%)	24,488 (85.2%)	48,953 (89.1%)	18,653 (76.9%)	14,962 (85.7%)	20,574 (80.9%)	6,537 (84.1%)	19,665 (83.8%)	317,160 (84.8%)
**TB status**	**tuberculosis + **	1,269 (1.1%)	938 (1.3%)	929 (3.2%)	927 (1.7%)	487 (2.0%)	200 (1.2%)	221 (0.9%)	140 (1.8%)	355 (1.5%)	5,466 (1.5%)
	**tuberculosis -**	116,925 (98.9%)	72,679 (98.7%)	27,813 (96.8%)	54,004 (98.3%)	23,771 (98.0%)	17,264 (98.9%)	25,217 (99.1%)	7,635 (98.2%)	23,115 (98.5%)	368,423 (98.5%)
**Facility type**	**hospital**	76,075 (64.4%)	50,028 (68.0%)	16,773 (58.4%)	25,472 (46.4%)	18,854 (77.7%)	14,299 (81.9%)	15,077 (59.3%)	4,406 (56.7%)	11,098 (47.3%)	231,909 (62.0%)
	**clinic**	42,119 (35.6%)	23,589 (32.0%)	11,969 (41.6%)	29,459 (53.6%)	5,404 (22.3%)	3,165 (18.1%)	10,361 (40.7%)	3,369 (43.3%)	12,372 (52.7%)	141,980 (38.0%)
**Year**	**2012**	29,381 (24.9%)	16,960 (23.0%)	9,606 (33.4%)	12,587 (22.9%)	8,249 (34.0%)	3,511 (20.1%)	6,147 (24.2%)	1,729 (22.2%)	5,614 (23.9%)	93,784 (25.1%)
	**2013**	33,277 (28.2%)	19,736 (26.8%)	9,654 (33.6%)	16,474 (30.0%)	6,359 (26.2%)	4,089 (23.4%)	6,820 (26.8%)	2,003 (25.8%)	6,389 (27.2%)	104,801 (28.0%)
	**2014**	39,159 (33.1%)	26,915 (36.6%)	7,764 (27.0%)	18,561 (33.8%)	6,581 (27.1%)	6,599 (37.8%)	9,538 (37.5%)	2,866 (36.9%)	8,115 (34.6%)	126,098 (33.7%)
	**2015**	16,377 (13.9%)	10,006 (13.6%)	1,718 (6.0%)	7,309 (13.3%)	3,069 (12.7%)	3,265 (18.7%)	2,933 (11.5%)	1,177 (15.1%)	3,352 (14.3%)	49,206 (13.2%)

*FBG* fasting blood glucose; *RBG* random blood glucose, *HbA1c* glycated hemoglobin A1c, *GP* Gauteng, *KZN* KwaZulu Natal, *WC* Western Cape, *EC* Eastern Cape, *FS* Free State, *LP* Limpopo, *MP* Mpumalanga, *NC* Northern Cape, *NW* North West

Supplemental Fig. 1a-j shows blood glucose and HbA1c testing events (patients could contribute > 1 test) in quarterly intervals stratified by health facility, HIV and tuberculosis status. In both hospital and clinics, the majority (68%; 1,063,137/1,562,255) of tests were HbA1c. We see an increase in use of HbA1c over the five-year period and an increase in both random and fasting blood glucose tests beginning around mid-2014 in all sub-groups and facility types. Around half (53%; 825,272/1,562,255) of testing for diabetes occurred in hospitals (Supplemental Fig. 1a-e). The number of patients tested using blood glucose at primary health care clinics was low (19%; 136,257/707,721).

### Diabetes care cascade

#### Overall

**Fig. 2:**
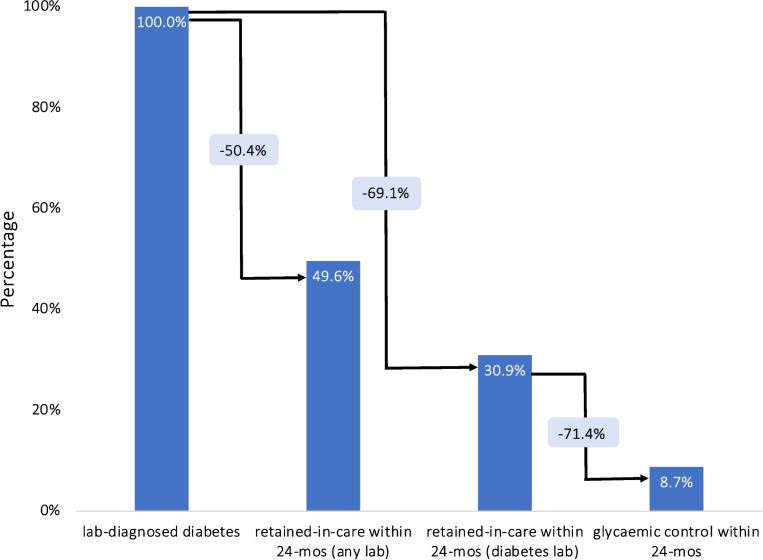
The diabetes care cascade based on laboratory data, South Africa 2012–2017 (*N* = 373,889) Denominator for the bars are total with lab-diagnosed diabetes and denominator for the light blue boxes is the previous stage

Figure 2 and Table 2 illustrate the laboratory diabetes care cascade in South Africa from 2012 to 2017 based on diabetes tests only. All presented estimates have been age-standardized. Out of 373,889 patients screened for diabetes, 186,664 (43.2%; 95% CI: 43.0–43.4%) had a lab value, HbA1c or blood glucose indicative of diabetes. Of those, 30.9% (95% CI: 30.7–31.2%) were retained-in-care, while only 8.7% (95% CI: 8.6–8.8%) achieved glycaemic control within 24 months after their lab diagnosis. When expanding the retained-in-care criterion to include all lab tests done within 24 months post-diagnosis, and not just diabetes-specific ones, a higher proportion of individuals with a lab-confirmed diabetes diagnosis sought care for other health concerns. Specifically, out of the 186,664 diagnosed individuals, 92,638 (49.6%; 95% CI: 49.4–49.9%) underwent any lab test in the following 24 months (Fig. 2). In contrast, only 60,624 pursued a diabetes-focused lab test in the same period, a difference of 32,014 patients. Most of these patients underwent tests for creatinine (32.3%), hemoglobin (24.1%), alanine transferase (10.8%), and tuberculosis GeneXpert (6.1%).

**Table 2:** The care cascade overall and transitions between the stages stratified by facility (hospital and clinic) and HIV status (*N* = 373,889)

	**No. patients tested** **n (%; 95% CI)**	**Lab-diagnosed diabetes** **n (%; 95% CI)**	**Retained-in-care within 24-mo** **n (%; 95% CI)**	**Glycaemic control within 24-mo** **n (%; 95% CI)**
National (*n* = 373,889)				
PLWH overall	56,729	16,286 (28.6%; 28.2–29.1%)	5,698 (34.3%; 33.4–35.1%)	1,859 (11.5%; 11.0–12.1%)
PLWOH overall	317,160	170,378 (47.3%; 47.1–47.5%)	54,926 (30.3%; 30.0–30.5%)	14,712 (8.3%; 8.2–8.4%)
Total overall	373,889	186,664 (43.2%; 43.0–43.4%)	60,624 (30.9%; 30.7–31.2%)	16,571 (8.7%; 8.6–8.8%)
PLWH transition between stages	56,729	16,286 (28.6%; 28.2–29.1%)	5,698 (34.3%; 33.4–35.1%)	1,859 (33.8%; 32.3–35.4%)
PLWOH transition between stages	317,160	170,378 (47.3%; 47.1–47.5%)	54,926 (30.3%; 30.0–30.5%)	14,712 (27.9%; 27.5–28.4%)
Total transition between stages	373,889	186,664 (43.2%; 43.0–43.4%)	60,624 (30.9%; 30.7–31.2%)	16,571 (28.6%; 28.1–29.0%)
Hospital (*n* = 231,909)				
PLWH overall	37,569	9,108 (24.7%; 24.2–25.2%)	2,664 (29.1%; 28.0–30.2%)	1,079 (11.8%; 11.1–12.5%)
PLWOH overall	194,340	90,680 (41.0%; 40.7–41.2%)	24,660 (26.1%; 25.8–26.4%)	8,509 (9.2%; 9.0–9.4%)
Total overall	231,909	99,788 (37.6%; 37.3–37.8%)	27,324 (26.6%; 26.3–26.9%)	9,588 (9.5%; 9.3–9.7%)
PLWH transition between stages	37,569	9,108 (24.7%; 24.2–25.2%)	2,664 (29.1%; 28.0–30.2%)	1,079 (40.7%; 38.3–43.2%)
PLWOH transition between stages	194,340	90,680 (41.0%; 40.7–41.2%)	24,660 (26.1%; 25.8–26.4%)	8,509 (35.4%; 34.7–36.2%)
Total transition between stages	231,909	99,788 (37.6%; 37.3–37.8%)	27,324 (26.6%; 26.3–26.9%)	9,588 (36.1%; 35.4–36.8%)
Clinic (*n* = 141,980)				
PLWH overall	19,160	7,178 (36.3%; 35.5–37.2%)	3,034 (41.3%; 39.8–42.8%)	780 (11.3%; 10.5–12.1%)
PLWOH overall	122,820	79,698 (60.0%; 59.6–60.4%)	30,266 (35.4%; 35.0–35.8%)	6,203 (7.2%; 7.0–7.3%)
Total overall	141,980	86,876 (53.8%; 53.4–54.1%)	33,300 (36.3%; 35.9–36.7%)	6,983 (7.6%; 7.4–7.8%)
PLWH transition between stages	19,160	7,178 (36.3%; 35.5–37.2%)	3,034 (41.3%; 39.8–42.8%)	780 (27.3%; 25.4–29.2%)
PLWOH transition between stages	122,820	79,698 (60.0%; 59.6–60.4%)	30,266 (35.4%; 35.0–35.8%)	6,203 (20.4%; 19.9–20.9%)
Total transition between stages	141,980	86,876 (53.8%; 53.4–54.1%)	33,300 (36.3%; 35.9–36.7%)	6,983 (21.2%; 20.7–21.7%)

*PLWH* People living with HIV,* PLWOH* People living without HIV

Transitions between stages of the diabetes care cascade are also displayed in Fig. 2 and Table 2. Estimates are conditional upon completion of the previous stage. Among those with lab-diagnosed diabetes, 30.9% (95% CI: 30.7–31.2%) were retained-in-care, while among those who were retained-in-care, 28.6% (95% CI: 28.1–29.0%) achieved glycemic control by 24 months.

#### Hospital vs. clinic

Table 2 also displays transitions between stages stratified by facility-type (hospital and clinic) based on diabetes tests only. Clinics tested a total of 141,980 individuals, out of which 53.8% (95% CI: 53.4–54.1%) were diagnosed with diabetes. This is notably higher than hospitals, which tested 231,909 individuals and diagnosed 37.6% (95% CI: 37.3–37.8%) of them. When observing retention-in-care post-diagnosis, clinics had a slightly higher estimate 36.3% (95% CI: 35.9–36.7%) compared to hospitals at 26.6% (95% CI: 26.3–26.9%). However, when considering diabetes control within 24 months, clinics reported 7.6% (95% CI: 7.4–7.8%), achieving glycemic control, which was slightly lower than the 9.5% (95% CI: 9.3–9.7%) achieved by hospitals. In terms of transitioning between stages, hospitals showed a higher proportion of patients achieving glycemic control at 36.1% (95% CI: 35.4–36.8%) compared to 21.2% (95% CI: 20.7–21.7%) in clinics.

#### PLWH vs PLWOH

Among the 317,160 PLWOH tested for diabetes, 47.3% (95% CI: 47.1–47.5%) received a positive diagnosis (Table 2). This rate was notably higher than the 28.6% (95% CI: 28.2–29.1%) diagnosed among the 56,729 PLWH. Upon diagnosis, PLWH exhibited better retention-in-care based on diabetes tests only, with 34.3% (95% CI: 33.4–35.1%) retained-in-care within 24 months, compared to 30.3% (95% CI: 30.0–30.5%) of PLWOH. When considering glycemic control within the same 24 month period, PLWH again demonstrated a slight advantage, with 11.5% (95% CI: 11.0–12.1%) achieving this health outcome versus 8.3% (95% CI: 8.2–8.4%) of PLWOH. As for the transition between care stages, a higher proportion of PLWH, 33.8% (95% CI: 32.3–35.4%), achieved glycemic control when compared to the 27.9% (95% CI: 27.5–28.4%) of PLWOH.

#### Acute tuberculosis infection vs. no tuberculosis infection

Among the 5,466 individuals diagnosed with acute tuberculosis infection, 32.6% (95% CI: 31.1–34.1%) were found to have diabetes. In contrast, of the 368,423 individuals without tuberculosis infection, a higher proportion of 43.4% (95% CI: 43.2–43.6%) were diagnosed with diabetes (Supplemental Table 1). Post-diagnosis, 25.1% (95% CI: 22.9–27.2%) of those with tuberculosis infection were retained-in-care over 24 months based on diabetes tests only, lower than what was observed in those without tuberculosis (31.0%; 95% CI: 30.7–31.2%). Glycemic control within 24 months was achieved by 6.9% (95% CI: 5.7–8.0%) of those with tuberculosis infection and 8.7% (95% CI: 8.6–8.9%) of those without. In the transition between stages of care, 26.2% (95% CI: 22.6–30.2%) of the tuberculosis infected group achieved glycemic control, slightly less than the 28.6% (95% CI: 28.1–29.0%) observed in those without tuberculosis.

#### Predictors of lab-diagnosed diabetes

Table 3 details the findings from the mixed effects Poisson regression. The data revealed that with advancing age, there's a higher likelihood of receiving a lab diagnosis for diabetes, with females showing a slightly elevated probability compared to males (adjusted Risk Ratio (aRR): 1.04; 95% CI: 1.02, 1.05). PLWH vs. PLWOH exhibited a 27% decreased risk (aRR: 0.73; 95% CI: 0.71–0.74) of lab-diagnosed diabetes. Those with an acute tuberculosis infection had a 5% increased risk (aRR: 1.05; 95% CI: 1.00–1.11) of a diabetes diagnosis, relative to their non-tuberculosis counterparts. Moreover, individuals screened at clinics, as opposed to hospitals, had a 7% greater likelihood (aRR: 1.07; 95% CI: 1.03–1.11) of a diabetes diagnosis. Risk of having a lab-diagnosis of diabetes decreased over time – by 3% in 2013, 8% in 2014 and 10% in 2015 when compared to 2012. Those diagnosed with diabetes via fasting plasma glucose and random plasma glucose tests were 49% and 74% less likely to have a lab-diagnosis of diabetes than those tested with HbA1c.

**Table 3:** Crude and adjusted nested models for the outcomes of laboratory-diagnosed diabetes, retained-in-care and glycaemic control in the national cohort (*N* = 373,889)

Characteristic	Measure	Lab-diagnosed diabetes		Retained-in-care		Glycaemic control	
		**Crude RR (95% CI)**	**Adjusted RR (95% CI)**	**Crude RR (95% CI)**	**Adjusted RR (95% CI)**	**Crude RR (95% CI)**	**Adjusted RR (95% CI)**
**Gender**	Male	ref	ref	ref	ref	ref	ref
	Female	1.05 (1.04, 1.06)	1.04 (1.02, 1.05)	1.05 (1.04, 1.07)	1.05 (1.03, 1.07)	0.85 (0.81, 0.88)	0.85 (0.82, 0.89)
**Age (years)**	30–39.9	ref	ref	ref	ref	ref	ref
	40–49.9	1.56 (1.53, 1.60)	1.31 (1.29, 1.34)	1.08 (1.04, 1.12)	1.08 (1.05, 1.12)	0.80 (0.73, 0.88)	0.85 (0.77, 0.93)
	50–59.9	1.83 (1.79, 1.86)	1.40 (1.37, 1.43)	1.14 (1.10, 1.18)	1.15 (1.11, 1.19)	0.86 (0.79, 0.93)	0.94 (0.86, 1.02)
	> 60	1.78 (1.74, 1.81)	1.32 (1.29, 1.34)	1.06 (1.03, 1.09)	1.07 (1.04, 1.11)	1.14 (1.05, 1.23)	1.27 (1.16, 1.38)
**HIV**	PLWOH	ref	ref	ref	ref	ref	ref
	PLWH	0.55 (0.54, 0.56)	0.73 (0.71, 0.74)	1.11 (1.08, 1.14)	1.12 (1.09, 1.15)	1.30 (1.22, 1.39)	1.42 (1.32, 1.52)
**Acute tuberculosis infection**	tuberculosis -	ref	ref	ref	ref	ref	ref
	tuberculosis +	0.77 (0.73, 0.81)	1.05 (1.00, 1.11)	0.87 (0.79, 0.95)	0.85 (0.78, 0.93)	0.85 (0.66, 1.09)	0.79 (0.61, 1.02)
**Facility type at first test**	Hospital	ref	ref	ref	ref	ref	ref
	Clinic	1.27 (1.21, 1.33)	1.07 (1.03, 1.11)	1.44 (1.34, 1.54)	1.41 (1.32, 1.51)	0.71 (0.66, 0.76)	0.73 (0.68, 0.78)
**Diagnostic used**	HbA1c	ref	ref	-	-	ref	ref
	FPG	0.48 (0.47, 0.49)	0.51 (0.50, 0.52)	-	-	2.13 (1.79, 2.54)	1.98 (1.65, 2.39)
	RPG	0.23 (0.23, 0.24)	0.26 (0.25, 0.26)	-	-	1.98 (1.69, 2.33)	1.75 (1.48, 2.07)
**Year**	2012	ref	ref	ref	ref	ref	ref
	2013	1.22 (1.20, 1.24)	0.97 (0.96, 0.99)	1.13 (1.10, 1.16)	1.13 (1.10, 1.16)	1.03 (0.97, 1.10)	1.05 (0.98, 1.12)
	2014	1.13 (1.11, 1.15)	0.92 (0.91, 0.94)	1.15 (1.13, 1.18)	1.15 (1.13, 1.18)	1.21 (1.14, 1.30)	1.23 (1.16, 1.31)
	2015	1.05 (1.03, 1.07)	0.90 (0.89, 0.92)	1.21 (1.17, 1.24)	1.21 (1.17, 1.24)	1.23 (1.14, 1.34)	1.24 (1.15, 1.35)

#### Predictors of retention-in-care (based on diabetes labs only)

Table 3 also illustrates that among patients diagnosed with diabetes, older individuals and females exhibited a greater propensity to be retained-in-care compared to their younger and male counterparts, respectively. PLWH showed an increased likelihood of staying in care (aRR 1.12; 95% CI: 1.09–1.15) in contrast to PLWOH. Those with an acute tuberculosis infection were less likely to be retained-in-care (aRR 0.85; 95% CI: 0.78–0.93) when compared to those without tuberculosis. Furthermore, patients diagnosed at clinics, as opposed to hospitals, displayed a notable 41% increased probability (aRR: 1.41; 95% CI: 1.32–1.51) of being retained-in-care. A trend was also observed regarding the year of entry into care. Compared to 2012, the likelihood of retention-in-care rose: by 13% in 2013, 15% in 2014, and 21% in 2015.

#### Predictors of glycaemic control

Table 3 highlights that among patients who were retained-in-care, individuals ≥ 60 years (when compared to the age group 30–39.9 years) had a higher likelihood of achieving glycemic control (aRR: 1.27; 95% CI:1.16–1.38). Conversely, those aged between 40–59.9 years, in comparison to the 30–39.9 age bracket, may have a diminished probability of reaching control (aRR: 0.85; 95% CI: 0.77, 0.93). PLWH, when compared to PLWOH, demonstrated a 42% increase in the probability of glycemic control (aRR: 1.42; 95% CI: 1.32, 1.52). However, patients with tuberculosis infection had a 21% reduced likelihood (aRR: 0.79; 95% CI:0.61, 1.02) of control compared to those without tuberculosis. In terms of facility type, individuals diagnosed at clinics, versus those at hospitals, had a 27% diminished likelihood of glycemic control. Moreover, female patients had a 15% decreased chance of control in comparison to their male counterparts. Notably, there was a consistent uptick in the probability of glycemic control from 2013 through to 2015 compared to 2012. Additionally, those diagnosed with diabetes via fasting plasma glucose and random plasma glucose tests had a 98% and 75% higher likelihood of achieving glycemic control than those tested with an HbA1c.

## Discussion

To our knowledge, our study is the first to assess the diabetes care cascade prospectively at a national level using South Africa’s NHLS database. Our assessment revealed major gaps in patients retention-in-care and reaching glycaemic control over 24-months post a diabetes lab-diagnosis, which is in line with previous research showing that diabetes care in sub-Saharan African countries remains poor [3, 4]. It is important to note that because we defined the steps of the care cascade prospectively in our study, our results differ from many of the more recent published cross-sectional studies assessing stages of the care cascade [3, 4], as by design cross-sectional studies cannot be used to assess incidence or temporal relationships between outcomes and risk factors.

We observed a surge in blood glucose tests around mid-2014, which likely aligns with the introduction of new primary health care guidelines. In our cohort, the prevalence of lab-diagnosed diabetes stood at 43%. This aligns with our expectations, given that our cohort likely represents a selective sample of patients exhibiting symptoms of, or at risk for, diabetes. While direct estimation of screening rates is not feasible from the lab data alone, the findings are suggestive. If almost half of the PLWOH and over a quarter of the PLWH with a diabetes lab test in the NHLS from 2012 to 2015 exhibit elevated blood glucose levels, it indicates that South Africa's current screening measures might be insufficient and that more screening could be beneficial.

In our research, of the patients with a laboratory-confirmed diabetes diagnosis, only 30.5% continued their care as indicated by at least one diabetes-specific lab test over a 24-month follow-up. Our observed attrition rate is higher than recent cross-sectional studies [3, 4], but it is consistent with findings from smaller regional prospective studies [14–17]. When we expand our definition of retention-in-care to include all lab tests—not solely those specific to diabetes—an alternate view emerges. Under this expanded criterion, 49.6% of patients were retained in the healthcare system. Notably, within this percentage, 34.6% did not partake in any follow-up diabetes-specific tests but were involved in other medical tests. Such patterns suggest potential gaps in consistent diabetes monitoring, possibly leaning towards point-of-care testing, and emphasize the need for deeper exploration of patients' healthcare engagements. Broadly in sub-Saharan Africa, a myriad of challenges plague every phase of the healthcare process [18]. Factors like societal and professional unawareness of diabetes, late diagnoses, insufficient diagnostic tools in clinics, and logistical hurdles like sample processing and transport, all play a part [19]. Specifically focusing on diabetes self-management, it often falls short of optimal [20], pushing some patients to discontinue treatment entirely. In South Africa, government-run facilities are the primary healthcare providers for a majority of diabetes patients. Yet, these facilities grapple with issues like overcrowding, resulting in extended wait times for patients during their monthly medication visits. Coupled with the financial burdens of diabetes care, the healthcare system faces considerable strain [21]. Given the multifaceted challenges and substantial attrition rates, it is crucial to implement targeted systemic changes to enhance patient retention throughout their care cascade journey.

Effective glycaemic control is essential to reduce diabetes complications yet many with diabetes in South Africa and the region fail to achieve adequate glycaemic control [3, 18]. In our study, among those with lab-diagnosed diabetes, only 8.7% had an HbA1c or blood glucose indicating glycaemic control within 24 months, which is within the range of estimates reported in the region (4.0% to 36.6%^3^). Our estimate is lower than what has been previously reported for South Africa (15 to 29%) [3, 4, 18, 19, 22, 23], however, most likely due to the majority of prior studies being cross sectional in nature, which would tend towards overestimating care cascade completion stages relative to longitudinal studies due to their inability to assess incidence.

Our analysis shows that PLWH had a 28.6% likelihood of testing positive for diabetes, significantly lower than the 47.3% observed in PLWOH. Existing research from sub-Saharan Africa on the interplay between HIV and diabetes is relatively scant, yielding diverse findings [21]. Notably, only a select number of studies have ventured a direct comparison between PLWH and PLWOH within this setting. Our observations align with research emanating from southern [24] and eastern [25] Africa, which report a diminished likelihood of a diabetes diagnosis in PLWH as opposed to PLWOH. Factors traditionally associated with diabetes risk, such as obesity and older age, appear less frequently in PLWH [26]. Additionally, it's worth noting a potential selection bias: younger individuals in their 20s or 30s seeking healthcare are more likely to have conditions like HIV, affecting the overall makeup of our clinic sample. Furthermore, the approach to diabetes lab testing for PLWH might be broader, reflecting perhaps a presumption of a heightened diabetes risk in this group.

The presence of multiple morbidities complicates patient care, especially when the diseases differ in pathogenesis and management. We found that PLWH had a lower risk of being diagnosed with diabetes through lab tests and were more likely to stay engaged in care. While, individuals with acute tuberculosis infections faced a higher risk of being diagnosed with diabetes but were less likely to be retained-in-care. This difference could be attributed to the fact that tuberculosis is typically a curable and often acute condition, while HIV requires ongoing, chronic care. Regardless, regular clinic visits for PLWH on antiretroviral therapy and those on tuberculosis treatment presents an opportunity for screening, diagnosis and treatment of diabetes. In South Africa, PLWH on antiretroviral therapy may have better managed diabetes than the general population, but evidence is lacking. In our cohort, there was a slightly higher proportion of PLWH compared to the general population in regards to retention-in-care. PLWH were more likely to have controlled diabetes 24-months post diagnosis compared to PLWOH, however the proportion was still very low. These results suggest the existing infrastructure of HIV care in South Africa has not been fully leveraged [27, 28] to improve screening, diagnosis, and treatment of non-communicable diseases. As testing and diagnosis of diabetes and other non-communicable diseases increases for PLWH and/or tuberculosis, it will be important to consider how to optimize care for patients with multiple co-morbidities. Additionally, the recent shift in treatment strategies, including the adoption of dolutegravir (known to cause excessive weight gain [29, 30] and an increased risk of hypertension [30]) as a first-line antiretroviral therapy, supplanting efavirenz (a weight-sparing agent), adds a new layer of complexity to the healthcare landscape.

The disparities in diabetes testing, diagnosis, and management between clinics and hospitals shed light on the nuances of care delivery across these healthcare infrastructures. With a diagnosis rate of 53.8% in clinics compared to 37.6% in hospitals, it is evident that clinics play a vital role in the primary healthcare sphere of South Africa, where most diabetes patients should be screened, diagnosed, and managed [31]. This underscores the central function of clinics in initial diagnosis and care. Despite clinics having superior patient retention rates at 36.3% post-diagnosis compared to hospitals at 26.6%, the achievement of glycemic control within both settings was suboptimal, falling below 10%. Such findings suggest that while clinics excel in early diagnosis and keeping patients in care, both settings grapple with the challenges of long-term diabetes management. The marked variances in transition rates across care stages accentuate the distinct strategies and existing care gaps between clinics and hospitals. These insights call for a comprehensive assessment and re-calibration of care frameworks, ensuring that both clinics and hospitals are poised to provide the best possible diabetes care.

Throughout 2013, 2014, and 2015, we observed a notable rise in lab-confirmed diabetes diagnoses, complemented by an increase in sustained patient care and more individuals achieving glycemic control, when contrasted with 2012 data. This positive trajectory can be largely attributed to South Africa's commitment to the Integrated Chronic Disease Management (ICDM) framework [32]. ICDM seeks to harness the innovative approaches from the HIV program, applying them to elevate the standard of care for all chronic conditions, diabetes included. This model underscores a comprehensive approach to patient care, prioritizing continuous monitoring and prompt interventions. By infusing proven strategies from HIV care into the broader chronic disease landscape, the ICDM has been pivotal in driving improvements in care quality and system effectiveness. The progress seen during these years is testament to the ICDM's impactful role in revolutionizing chronic disease care.

The primary strength of our study is the extensive size of our national cohort (*n* = 373,889). However, our data have important limitations. First, the probabilistic matching technique we employed to assemble our cohort had the potential for both over-matching and under-matching. This could result in either inflating or diminishing our outcome estimations. However, we did not address missing data, as our primary results excluded patients linked to laboratory results deemed unreliable. Even if these patients were factored in, the core findings would remain stable. For instance, if we postulate that under-matching errors were evenly distributed, our sensitivity analysis suggests that at the very most, we might be underestimating retention-in-care by 22% (1.0–0.78). Therefore, even an adjustment by 28% (1/0.78) to the estimations would not significantly modify our overarching conclusions. Second, since our assessment of the cascade starts with those screened for diabetes, we are unable to assess attrition in the cascade at the screening step and could be overestimating cascade performance relative to a study that allows for loss at the screening stage. As such, cascade completion rates could be worse than what we identified here. Moreover, we refrained from estimating screening rates in this analysis, believing that any such estimates would hinge heavily on broad assumptions. Third, our cohort may exhibit selection bias, specifically surveillance bias, since patients deemed eligible for analysis were likely targeted for preliminary diabetes testing at health facilities due to higher risk factors. Point-of-care testing, such as with glucometers, was typically used for initial screening. If elevated glucose levels were detected, blood samples would be sent to the NHLS for further confirmation. This approach is reflected in our cohort, where over 40% of patients were diagnosed with diabetes, a figure significantly higher than the estimated national prevalence of approximately 11.3% [2]. This discrepancy suggests that the patients selected for further testing may have been in a more advanced stage of diabetes, thus skewing our results. Although, it is important to reiterate that we were unable to identify the reason for testing in our cohort. Consequently, we cannot accurately estimate the population-level prevalence of diabetes using this data. However, this would only be a significant limitation if the screening were specifically based on factors related to retention-in-care. Fourth, a substantial number of patients in our study underwent diabetes testing in a hospital setting. However, we lack specifics regarding their exact diagnoses or reasons for testing in a hospital. A notable concern is stress hyperglycemia — a temporary spike in blood sugar. Such spikes can be mistakenly viewed as chronic diabetes, especially when aligned with acute conditions like active tuberculosis or other secondary infections post antiretroviral therapy initiation in PLWH, such as cryptococcal meningitis or non-tuberculosis pneumonia. This may result in an overestimation of diabetes prevalence in our cohort by confusing temporary glucose elevations with persistent metabolic issues. Fifth, our study focused on a cohort from 2012–2015 to describe the care cascade. Trends likely progressed beyond the time period of our study, with a pronounced transformation during and after the COVID pandemic, especially concerning the screening, monitoring, and treatment of diabetes. Finally, there is also the potential for uncontrolled confounding in our analysis, as the labs data lacks certain patient level clinical factors. Linkage of the laboratory data to patient level clinic data to obtain screening data and assess additional confounders would potentially improve the analysis.

## Conclusion

In this study, which utilized South Africa's NHLS database to assess the diabetes care cascade at a national level, we identified notable challenges in the diabetes care continuum. Specifically, 70% of patients diagnosed with diabetes did not have a diabetes specific follow-up lab, and less than 10% achieved glycaemic control within 24 months of diagnosis. In our cohort, diabetes prevalence differs between PLWH and PLWOH. Such variation might arise from differing screening approaches used for the two groups. While these disparities in diabetes prevalence introduce complexities, they also underscore opportunities within the current HIV care systems to enhance diabetes management. By examining the structured tracking and continuity in HIV care, we see potential templates for managing other chronic diseases, including non-communicable diseases. As the global community strives for Universal Health Coverage (UHC) by 2030, aligning with the United Nations' Sustainable Development Goals, it becomes imperative to tap into established practices from successful health programs, such as HIV care [33]. Doing so not only ensures efficient utilization of resources but also guarantees a high standard of care. Using such approaches can propel us closer to UHC while strengthening non-communicable disease management. In conclusion, our findings emphasize the need for tailored strategies to better screen, diagnose, and treat diabetes and other non-communicable diseases in South Africa, while remaining cognizant of the region's distinct challenges and nuances.

### Supplementary Information

**Additional file 1:** **Supplemental Figure 1a-j**. Quarterly glucose (random or fasting) and HbA1c lab events nationally and stratified by HIV status, tuberculosis status and facility type.

**Additional file 2:** **Supplemental Table 1. **The transitions between the stages in the diabetes care cascade stratified by facility (hospital and clinic) and tuberculosis status (*N*=373,889).

## Data Availability

The data in our study are not publicly available due to the terms of our contract with the NHLS, but are available from the NHLS with reasonable request.
