# Supplementary material for: Gaps in the type 2 diabetes care cascade: a national perspective using South Africa’s National Health Laboratory Service (NHLS) database
Source: BMC Health Serv Res. 2023 Dec 21;23:1452. doi: 10.1186/s12913-023-10318-9 (PMC10740239; doi:10.1186/s12913-023-10318-9)
Supplement: Supplementary file 1 — Additional file 1: Supplemental Figure 1a-j. Quarterly glucose (random or fasting) and HbA1c lab events nationally and stratified by HIV status, tuberculosis status and facility type. [file 12913_2023_10318_MOESM1_ESM.docx]

**Supplemental Figure 1a-1j. Quarterly glucose (random or fasting) and HbA1c lab events nationally and stratified by HIV status, tuberculosis status and facility type.**

**
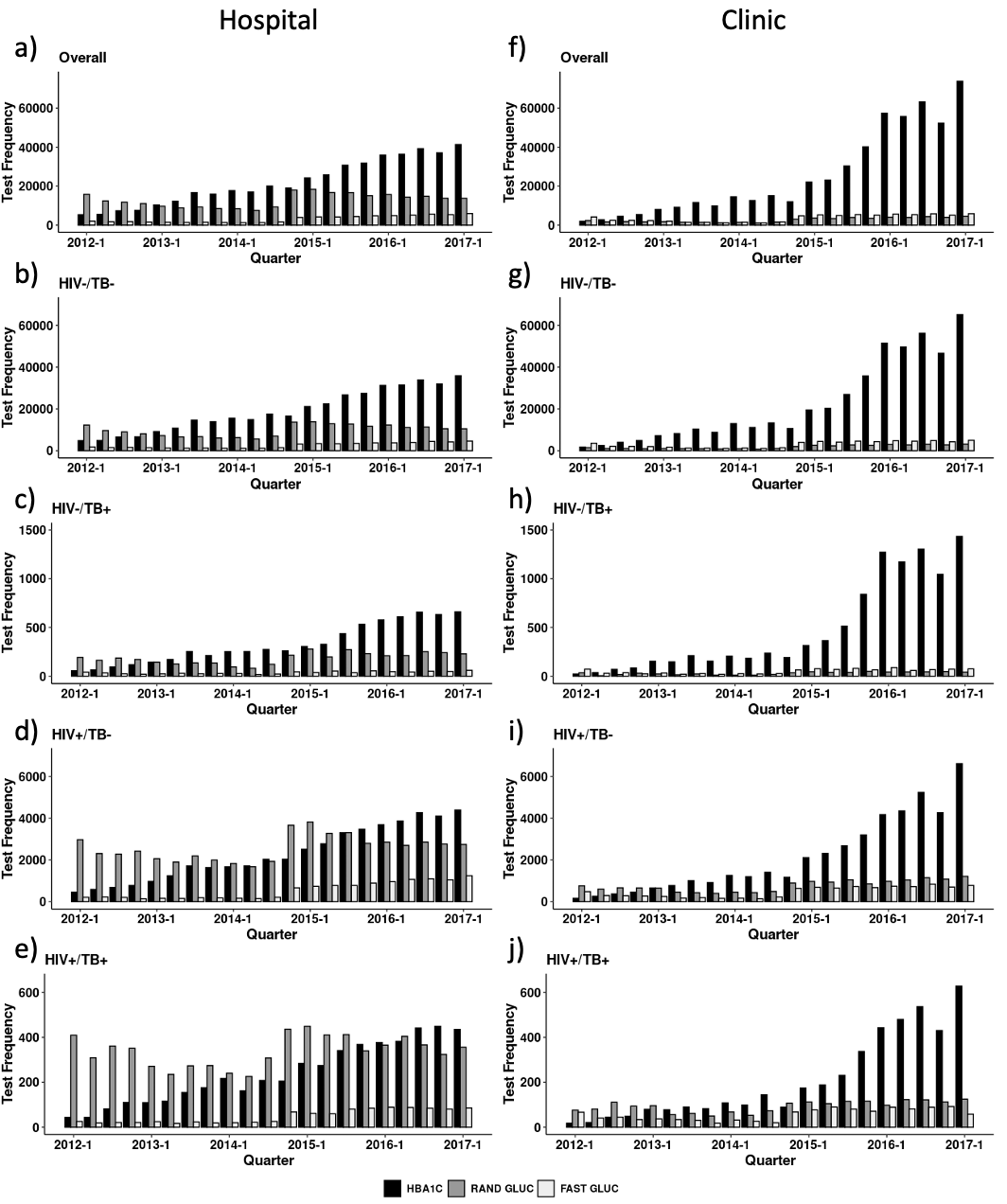
**
