# Supplementary material for: Gaps in the type 2 diabetes care cascade: a national perspective using South Africa’s National Health Laboratory Service (NHLS) database
Source: BMC Health Serv Res. 2023 Dec 21;23:1452. doi: 10.1186/s12913-023-10318-9 (PMC10740239; doi:10.1186/s12913-023-10318-9)
Supplement: Supplementary file 2 — Additional file 2: Supplemental Table 1. The transitions between the stages in the diabetes care cascade stratified by facility (hospital and clinic) and tuberculosis status (N=373,889). [file 12913_2023_10318_MOESM2_ESM.docx]

**Supplemental Table 1. The transitions between the stages in the diabetes care cascade stratified by facility (hospital and clinic) and TB status (N=373,889).**

|  |  | **No. patients**  **tested**  **n (%; 95% CI)** | | **Lab-diagnosed**  **diabetes**  **n (%; 95% CI)** | | **Remained in care**  **within 24-mo.**  **n (%; 95% CI)** | | **Controlled diabetes**  **within 24-mo.**  **n (%; 95% CI)** | |
| --- | --- | --- | --- | --- | --- | --- | --- | --- | --- |
| **National (n=373,889)** | | | | | | | | | |
| **TB+ Overall** | | | 5,466 | | 1,856 (32.6%; 31.1-34.1%) | | 507 (25.1%; 22.9-27.2%) | | 133 (6.9%; 5.7-8.0%) |
| **TB- Overall** | | | 368,423 | | 184,808 (43.4%; 43.2-43.6%) | | 60,117 (31.0%; 30.7-31.2%) | | 16,438 (8.7%; 8.6-8.9%) |
| **Total** | | | 373,889 | | 186,664 (43.2%; 43.0-43.4%) | | 60,624 (30.9%; 30.7-31.2%) | | 16,571 (8.7%; 8.6-8.8%) |
| **TB+ Transition Between Stages** | | | 5,466 | | 1,856 (32.6%; 31.1-34.1%) | | 507 (25.1%; 22.9-27.2%) | | 133 (26.2%; 22.6-30.2%) |
| **TB- Transition Between Stages** | | | 368,423 | | 184,808 (43.4%; 43.2-43.6%) | | 60,117 (31.0%; 30.7-31.2%) | | 16,438 (28.6%; 28.1-29.0%) |
| **Total** | | | 373,889 | | 186,664 (43.2%; 43.0-43.4%) | | 60,624 (30.9%; 30.7-31.2%) | | 16,571 (28.6%; 28.1-29.0%) |
| **Hospital (n=231,909)** | | | | | | | | | |
| **TB+ Overall** | | | 4,192 | | 1,265 (29.7%; 28.0-31.3%) | | 302 (22.0%; 19.5-24.5%) | | 90 (6.7%; 5.3-8.1%) |
| **TB- Overall** | | | 227,717 | | 98,523 (37.7%; 37.5-38.0%) | | 27,022 (26.7%; 26.4-27.0%) | | 9,498 (9.6%; 9.4-9.8%) |
| **Total** | | | 231,909 | | 99,788 (37.6%; 37.3-37.8%) | | 27,324 (26.6%; 26.3-26.9%) | | 9,588 (9.5%; 9.3-9.7%) |
| **TB+ Transition Between Stages** | | | 4,192 | | 1,265 (29.7%; 28.0-31.3%) | | 302 (22.0%; 19.5-24.5%) | | 90 (31.4%; 24.9-37.9%) |
| **TB- Transition Between Stages** | | | 227,717 | | 98,523 (37.7%; 37.5-38.0%) | | 27,022 (26.7%; 26.4-27.0%) | | 9,498 (36.2%; 35.4-36.9%) |
| **Total** | | | 231,909 | | 99,788 (37.6%; 37.3-37.8%) | | 27,324 (26.6%; 26.3-26.9%) | | 9,588 (36.1%; 35.4-36.8%) |
| **Clinic (n=141,980)** | | | | | | | | | |
| **TB+ Overall** | | | 1,274 | | 591 (41.6%; 38.2-45.0%) | | 205 (32.6%; 28.2-37.1%) | | 43 (6.9%; 4.8-8.9%) |
| **TB- Overall** | | | 140,706 | | 86,285 (53.9%; 53.6-54.3%) | | 33,095 (36.4%; 36.0-36.7%) | | 6,940 (7.6%; 7.4-7.8%) |
| **Total** | | | 141,980 | | 86,876 (53.8%; 53.4-54.1%) | | 33,300 (36.3%; 35.9-36.7%) | | 6,983 (7.6%; 7.4-7.8%) |
| **TB+ Transition Between Stages** | | | 1,274 | | 591 (41.6%; 38.2-45.0%) | | 205 (32.6%; 28.2-37.1%) | | 43 (23.3%; 16.4-30.3%) |
| **TB- Transition Between Stages** | | | 140,706 | | 86,285 (53.9%; 53.6-54.3%) | | 33,095 (36.4%; 36.0-36.7%) | | 6,940 (21.2%; 20.7-21.6%) |
| **Total** | | | 141,980 | | 86,876 (53.8%; 53.4-54.1%) | | 33,300 (36.3%; 35.9-36.7%) | | 6,983 (21.2%; 20.7-21.7%) |
